# A Comparative Study of Characteristics and Outcomes of Patients with Proved and Suggested Sarcoid Uveitis Occurring after Ophthalmic Procedure

**DOI:** 10.1155/2018/2954546

**Published:** 2018-10-18

**Authors:** Yvan Jamilloux, Aude Taleb, Audrey De Parisot, Laurent Pérard, Carole Burillon, Christiane Broussolle, Laurent Kodjikian, Pascal Sève

**Affiliations:** ^1^Department of Internal Medicine, Hôpital de la Croix Rousse, Hospices Civils de Lyon, Université Claude Bernard Lyon 1, Lyon, France; ^2^Department of Ophthalmology, Hôpital de la Croix Rousse, Hospices Civils de Lyon, Université Claude Bernard Lyon 1, Lyon, France; ^3^Department of Internal Medicine, Hôpital Edouard Herriot, Hospices Civils de Lyon, Université Claude Bernard Lyon 1, Lyon, France; ^4^Department of Ophthalmology, Hôpital Edouard Herriot, Hospices Civils de Lyon, Université Claude Bernard Lyon 1, Lyon, France

## Abstract

**Purpose:**

To describe patients with new onset sarcoid uveitis occurring after an ophthalmic procedure and compare them with patients with sarcoid uveitis without ocular procedure.

**Methods:**

Retrospective analysis of case records from patients with postophthalmic procedure sarcoid uveitis seen at our institution between April 2004 and October 2016. Patients with a previous history of uveitis were not included. Each patient was randomly matched with four controls from our incident cohort of new onset sarcoid uveitis without ophthalmic procedure.

**Results:**

We identified 11 patients (8.5%) from our incident cohort of sarcoid uveitis (*n*=130), who were all women, with a postophthalmic procedure uveitis (mostly after cataract surgery (36%)). These patients were older (69.7 vs 52.7 years) and presented more synechiae than controls. After a mean follow-up of 30 (3–60) months, there was no significant difference between the postprocedure and the control group with regard to demography, clinical presentation, disease course, treatment, and outcome.

**Conclusions:**

Sarcoid uveitis has similar characteristics in patients with new onset sarcoid uveitis after or without ophthalmic procedure. As a consequence, ophthalmic intervention should be seen as a potential trigger of latent sarcoidosis.

## 1. Introduction

Ocular surgery may be responsible for new onset uveitis as a part of postcataract surgical inflammation [1]. Other types of chronic inflammation (defined by a duration >6 weeks) can occur after an ocular surgery: endophthalmitis [2] (the most severe), or Irvine–Gass syndrome (the most frequent) [3] or sympathetic ophthalmia [4]. Other acute inflammatory reactions (duration <6 weeks) can occur after ophthalmic procedure, such as phacoantigenic uveitis, toxic anterior segment [5] syndrome, or toxic endothelial cell destruction [6].

Sarcoidosis is a systemic inflammatory disorder of unknown etiology, characterized by noncaseating epithelioid-cell granulomas, which primarily affects the lungs and the lymphatics. The spectrum of ocular manifestations is wide and highly variable; almost any part of the eye and other orbital structures can be affected [7, 8]. Ocular involvement may coexist with symptomatic or asymptomatic systemic disease manifestations, but may precede systemic involvement by several years [7, 8]. In retrospective series of biopsy-proven sarcoidosis, symptomatic uveitis affects 20–50% of patients [8], with 80% of these cases being diagnosed within the first year, of which 30% have uveitis as a presenting manifestation [7]. Uveitis is the most frequent ocular manifestation and may affect up to 20–30% of sarcoidosis patients [1].

Because patients can present with new onset sarcoid uveitis occurring after an ophthalmic procedure, we aimed at describing such cases and comparing them with patients with sarcoid uveitis without ocular procedure.

## 2. Patients and Methods

### 2.1. Patients

Case records of patients who were seen at one of the two departments of Internal Medicine and at one of the two departments of Ophthalmology of the Lyon University Hospitals between April 2004 and October 2016 were analyzed retrospectively.

Patients were included if

- uveitis occurred no more than one year after ophthalmic procedure;
- a biopsy analysis showed noncaseating granuloma or if patient met the Abad's modified criteria [9, 10]. According to these criteria, patients had *presumed* sarcoidosis if they had at least two of the following four criteria: typical changes on a chest radiograph or chest computed tomography (CT), predominantly CD4 lymphocytosis on bronchoalveolar lavage (BAL) fluid analysis, elevated serum angiotensin converting enzyme (ACE) levels, or a high gallium or 18F-labelled deoxyglucose positron emission tomography (^18^FDG-PET) uptake. If only one criterion was fulfilled, it was considered as a *possible* sarcoidosis.

Patients with a previous history of uveitis, those who had uveitis before ocular procedure, and those with other possible causes of uveitis (including endophthalmitis [2] and Irvine–Gass syndrome [11] with isolated macular edema on OCT and/or on fluorescein angiogram without any other inflammatory signs such as vitritis and vasculitis) were not included.

### 2.2. Control Group

Patients with postophthalmic procedure uveitis were compared with control subjects presenting sarcoid uveitis but no history of ophthalmic procedure. Patients in the control group were randomly selected using a computerized algorithm among the 130 incident cases of sarcoid uveitis recorded in the Lyon University Hospitals. Each patient with postophthalmic procedure sarcoid uveitis was matched with 4 controls.

### 2.3. Ethical Statement

The institutional review board of the *Hospices Civils de Lyon* gave its approval to retrospectively review the records. According to the French law (no. 2004-806, August 9, 2004) and because the data were collected retrospectively and patient management was not modified, this study did not require further research ethics committee approval.

### 2.4. Data Collection

Clinical and laboratory data were collected and analyzed using a standardized form: medical history, presenting symptoms, ophthalmic and systemic manifestations, sites of tissue biopsies, C-reactive protein (CRP), blood lymphocytes count, liver tests, calcemia, and serum ACE at diagnosis. Chest X-rays were rated according to the Scadding criteria. Results of other imaging findings (including chest CT), pulmonary function tests, bronchoscopies with BAL, cardiac investigations, detailed medical treatments, and disease outcome were also recorded when available.

### 2.5. Ophthalmic Examination

Ophthalmologic data, which included assessment of the best-corrected visual acuity (BCVA), intraocular pressure, complete biomicroscopic examination and optical coherence tomography (OCT), and fluorescein and indocyanine green angiographies, were collected at diagnosis and during follow-up by ophthalmologists. Uveitis was classified according to the Standardization of Uveitis Nomenclature (SUN) [12].

### 2.6. Statistical Analysis

Continuous variables were expressed as means (±standard deviation) and compared between groups using a Mann–Whitney test. Categorical variables were expressed as percentages and compared between groups with a Chi-square test or Fisher's exact test as appropriate. *P* values were considered significant when they were <0.05. Statistical analysis was performed with the R software (version 3.4.5, the R company).

## 3. Results

### 3.1. Case Reports

Detailed case reports are supplied in the Supplementary Materials (available here).

### 3.2. Global Characteristics of Patients

Among the 130 incident cases of sarcoid uveitis seen at our institution, we identified 11 patients (8.5%) who were diagnosed after an ophthalmic procedure. The patients' general characteristics are summarized in Table 1. All patients were women, with a mean age of 69 (27–88) years. Ocular procedures included cataract surgery (*n*=4), YAG capsulotomy (*n*=2), vitreoretinal surgery (*n*=2), trabeculectomy (*n*=1), vitreous injection (*n*=1), and laser therapy (*n*=1). No sign or sequelae of intraocular inflammation existed before ophthalmic procedure. The mean delay between the ophthalmic procedure and uveitis was 3.6 months (1–12 months).

Sarcoidosis was histologically proven in seven patients (minor salivary glands biopsy, *n*=2; fine-needle aspiration of mediastinal nodes, *n*=2; conjunctival nodule biopsy, *n*=2; and gastric biopsy, *n*=1).

Ophthalmologic findings consisted of chronic (*n*=10), bilateral (*n*=6), granulomatous (*n*=8) uveitis involving the anterior segment (*n*=3), the vitreous (*n*=1), and the retina or choroid (*n*=1), or a panuveitis (*n*=7). One patient had elevated intraocular pressure. Five patients had unilateral uveitis, but only three had an ophthalmic procedure performed on the symptomatic eye. In our tertiary center, preoperative chest X-ray is not performed routinely for patients undergoing cataract surgery. Thoracic imaging performed at the time of diagnostic workup showed abnormalities suggestive of sarcoidosis in nine cases: chest X-ray (*n*=1/11), chest CT scan (*n*=9/11), and nuclear imaging (*n*=8/11).

All patients had postoperative inflammation. Eight patients received topical steroids and six required a systemic treatment by steroids for a mean duration of 14 (0–30.4) months. Other therapies were needed in two patients (methotrexate and hydroxychloroquine). Among the five patients who still had a treatment at last visit, four were not symptomatic anymore. Treatments and visual prognoses are shown in Table 2.

### 3.3. Comparison of Postophthalmic Procedure Patients with Controls

Both groups were statistically similar in terms of gender, ethnicity, chest CT imaging results, and the other organs involvement. As a consequence of random matching, patients were older in the study group than in the control group. The prevalence of ophthalmic signs varied according to the group considered, with more synechiae and a trend for more granulomatous uveitis in the postprocedure group. Otherwise, there were no significant differences between groups with regard to the presence of an ocular hypertony, multifocal choroiditis, CSME, vasculitis, and papilledema. The groups were no different in terms of follow-up and in terms of recovery or treatment at the last visit (Table 3).

## 4. Discussion

To our knowledge, the present study is the first series of patients with postophthalmic procedure sarcoid uveitis and the first case-control series comparing these patients with patients without ocular intervention. In our tertiary center, postoperative sarcoid uveitis represents 8.5% of our sarcoid uveitis cohort.

New onset uveitis is one of the potential causes of postoperative inflammation occurring after a cataract surgery. This diagnosis is considered in patients with chronic postoperative inflammation, in which etiologies include chronic endophthalmitis and lens-related inflammation [7, 8]. Postoperative recurrence of uveitis in uveitic patients is a well-known complication [3, 13, 14]. Anterior chamber inflammation is also a potential side effect after a trabeculoplasty [15]. Anterior chamber inflammation may also be seen in 0.4–33.5% of patients after capsulotomy, but persistent vitritis is rare and no complication has been reported [16]. To avoid such bias, we excluded all patients with a previous history of uveitis and all patients had a fundus examination before eye procedures.

Sarcoidosis etiology remains unknown but several infectious agents have been postulated as triggering factors [17]. However, there is no definite evidence that sarcoidosis is an infectious disease; rather, sarcoidosis is considered as an exaggerated immune response to pathogen-associated molecular patterns of killed and/or partly degraded (myco) bacteria which persist within immune cells and resist degradation [17, 18]. For example, some patients have been reported as developing sarcoidosis after cardiac [19] or bone marrow transplantations [20, 21], suggesting the transmission of a potential activator of the disease. Therefore, we hypothesize that ophthalmic procedures may act as a trigger of (latent) sarcoidosis. The fact that systematic fundus examination was performed before eye procedures and did not detect signs of sarcoidosis, along with the occurrence of bilateral and contralateral sarcoid uveitis, reinforces such an hypothesis.

Our results show that ethnic distribution, laboratory features, treatment, and ophthalmologic outcome were similar in sarcoid uveitis patients after an ophthalmologic procedure or spontaneously. Nevertheless, postophthalmic procedure sarcoid uveitis seemed to affect more frequently women than men. This sex ratio in sarcoid uveitis has already been reported in other larger studies, yet not so predominantly [8, 22–24]. We also noticed an older age at onset in the postprocedure group. This can be due to the random matching of patients or because the number of patients who need an ocular procedure tends to increase over age. The number of Caucasian patients in our series was also higher than that in other European studies [24–26]. Finally, patients with postophthalmic procedure sarcoid uveitis had significantly more synechiae. This result was in line with a previous series of 281 biopsy-proven sarcoidosis patients, in which 20–26% had posterior synechiae [13]. Although Evans et al. have reported that two-thirds of sarcoid uveitis was not granulomatous, we noted more granulomatous precipitates in the postprocedure group than in the control group [25]. This suggests that surgery/procedure increases the intraocular inflammation, as this is the case in the well-known postsurgical Irvine–Gass uveitis [27, 28]. Drancourt et al. also reported the case of a postcataract surgery uveitis that led to the diagnosis of Whipple disease [29]. Therefore, we can imagine that the ocular procedure may act as a trigger of inflammation in patients with latent sarcoidosis. Two patients were affected bilaterally. There is no evident explanation, but we can relate this situation with the physiopathology of sympathetic ophthalmia. Sympathetic ophthalmia is a rare bilateral granulomatous uveitis due to trauma or surgery in one eye [30]. The etiology is not well elucidated but might be secondary to the development of an autoimmune reaction to ocular antigens exposed during the traumatic or surgical event with T cells as primary mediators [31]. The main hypothesis is an immune dysregulation with an autoimmune inflammatory response against ocular self-antigens. A similar mechanism of autoimmune dysfunction in sarcoidosis might explain bilateral uveitis in our two patients.

Chest imaging was suggestive of sarcoidosis in 12.5% of chest X-rays and 82% of chest CTs with no statistical difference between groups. These data support the results of previous series and suggest that chest CT should be performed in patients with negative chest X-rays while sarcoidosis is highly suspected [32–34].

Finally, our study has some limitations. It is difficult to establish precisely the delay between the procedure and the onset of uveitis. We chose a maximum delay of 12 months between the ocular procedure and the onset of symptoms since chronic postoperative endophthalmitis may appear insidiously several months after an ophthalmic procedure. The design of the study, with single center and retrospective inclusions, is also an important limitation that will require further studies to confirm our findings.

## 5. Conclusions

We describe herein the first series of postophthalmic procedure sarcoid uveitis. The major message is that sarcoid uveitis has similar characteristics (demography, course, and outcome) in patients with or without ophthalmic procedure. As a consequence, ophthalmic intervention should be seen as a potential trigger of latent sarcoidosis, especially in older Caucasian females.

## Figures and Tables

**Table 1:** Clinical, ophthalmic, radiologic, and histopathologic features of patients with postophthalmic procedure sarcoid uveitis.

Patient#	Sex	Age	Ophthalmic procedure	Uveitis							Systemic involvement	Sarcoidosis diagnosis
				Timeline	Unilateral/OU	Anatomical form	Granulomatous	Vasculitis	Choroiditis	Edema		
1	F	69	Cataract	1	OU	Pan	+	−	−	Macular		MSG, bronchial, gastric biopsies
2	F	76	Cataract	3 + 10	OU	Pan	−	−	−	Macular		*Negative*
3	F	82	Cataract	12	OU	Pan	+				Conjunctival nodule	Conjunctival nodule biopsy
4	F	31	Preretinal haemorrhage + macular hole	2	OU	Pan	+	+/+	−	ERM + papilledema		MSG biopsy
5	F	67	Glaucoma	5	OU	Pan	+EIOP	−	+	Macular	Sicca syndrome + conjunctival nodule weight loss	Gastric biopsy
6	F	81	Cataract	1	OU	A/I	+	−	−	Macular	Dyspnea	Positive ^18^FDG-PET
7	F	69	Retinal detachment	8	Unilateral	Pan	+	Venous	+			Abad's criteria
8	F	88	Cataract + YAG capsulotomy	1	Unilateral	I/P	+	−	−	Macular	Cough	Bronchial biopsy
9	F	83	Intravitreal aflibercept	1	Unilateral	A	+	−	−			*Negative*
10	F	70	Membrane peeling	5	Unilateral	A					Cough	Lymph node fine needle aspiration
11	F	58	YAG capsulotomy	4	Unilateral	I/P	−	−	−	Macular papilledema	Conjunctival nodule	Conjunctival nodule biopsy

F = female, age in years, OU = both eyes, A = anterior, I = intermediate, P = posterior, Pan = panuveitis, EIOP = elevated intraocular pressure, ERM = epiretinal membrane, ADP = adenopathy, MSG = minor salivary glands, and ^18^FDG-PET = ^18^F-labelled deoxyglucose positron emission tomography.

**Table 2:** Treatment of patients with postophthalmic procedure sarcoid uveitis.

Patient	Initial treatment	Outcome of sarcoidosis after initial treatment	Adverse effects of treatment	Subsequent treatment	Final outcome		Final visual acuity		Treatment at the end of follow-up	Follow-up duration (months)
					Ophthalmic outcome	Other manifestations	OR	OS		
1	Topical CS	CSME OS	No	Systemic CS	Atrophic MFC	Pulmonary nodules progression on CT scan	20/20	20/25	Systemic CS 5 mg/day	52
2	DXM intravitreal implant		No	Systemic CS	CSME		20/50	20/25	Systemic CS 1 mg/day	36
3	DXM intravitreal implant	CSME OR with vitreomacular traction	No	Pars plana vitrectomy with membrane peel	No CSME		0	20/200	No	3
4	Systemic CS 70 mg/day	Retinal vasculitis OU CSME papilledema	No	No	ERM, and no inflammation		20/20	20/25	Systemic CS 4 mg/day	37
5	Topical + systemic CS 20 mg/day		Osteopenia, skin fragility cushing-like syndrome, and CS dependency	No	Persistent bilateral KP, and no posterior inflammation		20/40	20/40	Topical CS	60
6	Topical + systemic CS 20 mg/day	Complete response several relapses: Vitritis OU	Cushing-like syndrome, osteopenia, skin fragility, and CS dependency	MTX	Anterior and intermediate inflammation	Pulmonary embolism	20/20	20/20	Systemic CS 9 mg/day + MTX 10 mg/week	53
7	Topical CS	Vitritis OR peripheral diffuse choroiditis atrophy	No	Systemic CS 40 mg	Vitritis OR persistent		—	—	Subconjunctival injections of triamcinolone	13
8	DXM intravitreal implant	Improvement of CSME	No	No	No inflammation		20/20	20/25	No	15
9	Topical CS	Complete response	No	No	No inflammation				No	19
10	Topical CS	Complete response	No	None	Papilledema	Chest pain	0	20/20	No	11
11	Topical CS	Complete response	No	Dexamethasone intravitreal implant	Recurrent CSME		20/50	20/25		40

CSME = Macular edema, OD = right eye, OS = left eye, OU = both eyes, MFC = multifocal choroiditis, ERM = epiretinal membrane, CT = computed tomography, MTX = methotrexate, and CS = corticosteroids.

**Table 3:** Comparison of postprocedure patients with control patients: demographic characteristics and ophthalmic, laboratory, and radiologic features.

	Patients withocular surgery (*n*=11)	Controls (*n*=44)	*P* value
Median age at diagnosis (years)	69.7	52.7	**0.004**
Females	11	32	**0.05**
Ethnicity Cau/Afr/North-Afr/As	10/1/0/0	35/1/7/1	0.33

*Ophthalmologic findings*			
Unilateral/bilateral/unknown	5/6/0	11/32/1	0.42
Granulomatous	9	20	0.07
Synechiae	1	20	**0.05**
Elevated intraocular pressure	1	2	0.26
Retinal vasculitis	2	5	0.50
Macular edema	8	18	0.16
Papilledema	2	6	0.51
Multifocal choroiditis	3	14	0.999
Other ophthalmic features at diagnosis	3	5	0.33
Other systemic features at diagnosis	2	11	1
Other systemic features during course	0	6	0.22

*Chest imaging*			
X-ray staging I/II/III/IV (scadding)	7/1/3/0	23/17/3/1	0.08

*Serum markers*			
Elevated ACE	7	18	0.65
Elevated lysozyme	6	22	0.999

*Treatment and outcome*			
Topical steroids	8	35	0.27
Systemic steroids	6	25	0.27
Immunosuppressants	1	12	0.12
Evolution to remission	5	13	0.76

Cau = Caucasian, Afr = African, North-Afr = North African, As = Asian, and ACE = angiotensin-converting enzyme.

## Supplementary Materials

Supplementary Materials Detailed case reports of the 11 patients who were diagnosed with sarcoid uveitis after an ophthalmic procedure.
