# Supplementary material for: A Comparative Study of Characteristics and Outcomes of Patients with Proved and Suggested Sarcoid Uveitis Occurring after Ophthalmic Procedure
Source: J Ophthalmol. 2018 Oct 18;2018:2954546. doi: 10.1155/2018/2954546 (PMC6211203; doi:10.1155/2018/2954546)
Supplement: Supplementary Materials — Detailed case reports of the 11 patients who were diagnosed with sarcoid uveitis after an ophthalmic procedure. [file 2954546.f1.doc]

**SUPPLEMENTARY MATERIAL**

# *Detailed case reports*

# *Case 1*

A 69-year-old African woman, with medical history of high blood pressure, diabetes mellitus, dyslipidemia and Parkinson’s disease, was admitted in December 2011 for a bilateral anterior and intermediate uveitis, one month after a bilateral phacoemulsification. The patient presented with granulomatous keratic precipitates (KPs) and a clinically significant macular edema (CSME). The chest CT scan showed bilateral pulmonary nodules. Minor salivary glands (MSG) and transbronchial biopsies confirmed the diagnosis of sarcoidosis. Topical steroids were administered for five months with a complete resolution of ocular symptoms. Later, in July 2016, the patient complained of a decreased visual acuity since one month and ophthalmic examination revealed bilateral multifocal choroiditis. A systemic steroid therapy was started. At last visit, in October 2016, under 5 mg/day of prednisone, the patient had only sequelae of choroiditis without signs of active inflammation.

***Case 2***

A 76-year-old Caucasian woman was admitted in November 2011 with a bilateral panuveitis, three months after a cataract surgery. On admission, examination found diffuse KPs and a CSME. The CT scan showed mediastinal lymphadenopathy and the BAL fluid analysis revealed alveolitis with a CD4/CD8 lymphocytes ratio of 5.8. MSG biopsy and spirometry were normal. A transbronchial biopsy showed non-caseating epithelioid granulomas consistent with sarcoidosis. After three partially efficient steroid intra-vitreous injections, the patient received a systemic steroid therapy. In March 2015, prednisone had been tapered to 7.5 mg/day, and there were no signs of CSME recurrence. At last visit, in January 2016, the examination showed no ophthalmic inflammation, hence prednisone was slowly weaned over 9 months.

***Case 3***

An 82-year-old Caucasian woman was referred to our institution for a bilateral panuveitis in April 2006. Her medical history included breast cancer, diabetes mellitus, central retinal vein occlusion, and glaucoma surgery. Moreover, she underwent cataract surgery less than one year before. On examination, a granulomatous bilateral panuveitis was noted, associated with a conjunctival nodule. The chest CT revealed mediastinal lymphadenopathy and ground glass opacities. A conjunctival biopsy confirmed the diagnosis of sarcoidosis. Uveitis spontaneously healed, and the patient was lost to follow-up.

***Case 4***

A 31-year-old Caucasian woman presented with a bilateral panuveitis, two months after a surgery for a macular hole and a preretinal haemorrhage. Ophthalmic examination revealed granulomatous KPs, a retinal vasculitis, along with a macular and papillary edema. The patient also complained of dyspnoea. The CT scan showed lymphadenopathy and pulmonary nodules. A MSG biopsy confirmed the diagnosis of sarcoidosis. The patient was treated with a systemic steroid therapy at 60 mg/day, which was tapered over 36 months. Clinical improvement was obtained and the patient is currently receiving 4 mg/day of prednisone.

***Case 5***

A 67-year-old Caucasian woman underwent a glaucoma surgery in July 2009. Five months after the trabeculotomy, the patient presented with bilateral acute panuveitis associated with an eight-kilograms weight loss. Ophthalmic examination showed KPs, ocular hypertension, and patchy sheathing multifocal choroiditis. Sicca syndrome and inferior conjunctival nodule were also noticed. The chest CT showed bilateral mediastinal lymphadenopathy. The 18-FDG PET revealed multiple hypermetabolisms of the submaxillar glands, mediastinal lymph nodes, and parotid glands.

Considering the weight loss, a gastroscopy was performed, and the biopsy was consistent with sarcoidosis. The patient received topical and systemic steroids. In January 2016, the ophthalmic examination showed persistent bilateral KPs with no posterior inflammation, under topical steroids alone.

***Case 6***

An 81-year-old Caucasian woman presented with a bilateral anterior and intermediate uveitis, one month after cataract surgery. Her previous medical history included breast cancer, mitral valve disease, hypothyroidism and osteoarthritis. Her ocular history included a pseudoexfoliative glaucoma, which was treated by timolol and dorzolamide eye drops. The ophthalmic examination showed bilateral granulomatous KPs and a left CSME. The chest X-ray and CT were normal but 18-FDG PET showed hypermetabolic mediastinal lymph nodes. The patient was treated with topical and systemic steroids (20 mg/day). Although the treatment was initially efficient, the patient developed a steroid dependence at 8 mg/day as well as steroids side effects (osteopenia, skin fragility and cushing-like syndrome). Therefore, methotrexate was added in March 2015, but the patient underwent several relapses. Eventually, cotton-wool nodules and perivenous infiltrates downsized with steroid dosage increases. At last visit, in April 2016, the examination revealed a recurrence of anterior and intermediate inflammation despite 10 mg/weeks of methotrexate and 9 mg/day of prednisone.

***Case 7***

A 69-year-old Caucasian woman presented with a unilateral panuveitis eight months after a laser therapy for peripheral retinal tears. The initial examination showed a chronic granulomatous, non–hypertensive, unilateral panuveitis with a multifocal choroiditis and a retinal vasculitis but there were no macular edema nor papilledema. The 18-FDG PET showed hypermetabolic parotid glands. ACE was also elevated (69.8 U/L). According to Abad’s modified criteria^9^, the diagnosis of possible sarcoidosis was considered. The patient received topical steroids and 40 mg/day of systemicsteroids, but did not recover. A treatment with three subconjunctival injections of triamcinolone was then started but the patient was lost to follow-up.

***Case 8***

An 88-year-old Caucasian woman was admitted to our hospital because of an episode of unilateral anterior uveitis, one month after a YAG capsulotomy. She had a past history of blood hypertension, diabetes mellitus, and dyslipidaemia. In 2006, a macular hole surgery followed by a cataract extraction was performed on the left eye. A phacoemulsification was performed in 2010 on the right eye. On admission, she had a unilateral intermediate and posterior uveitis, a CSME and a papilledema but no systemic involvement. The CT scan, the BAL fluid analysis and the 18-FDG PET were suggestive of sarcoidosis, which was confirmed by a bronchial biopsy. The patient was treated with 3 intravitreous dexamethasone injections. In October 2015, she had no active inflammation and the visual acuity was stable.

***Case 9***

An 83-year-old Caucasian woman underwent intravitreous injections for an exudative age-related macular degeneration from 2010 to March 2014 (ranibizumab, n=29 and aflibercept, n=2). One month later, the patient presented with a unilateralpainful eye and she was diagnosed with a first episode of granulomatous anterior uveitis. Ophthalmologic examination revealed granulomatous KPs and synechiae. The chest X-ray was normal but the chest CT showed bilateral mediastinal lymphadenopathy. Sarcoidosis was *possible* according to Abad’s criteria. One year later, the follow-up examination showed no ocular inflammation.

***Case 10***

A 70-year-old Caucasian woman underwent a vitreoretinal surgery for an epiretinal membrane. She had a medical history of diabetes mellitus, hypertension, and dyslipidemia. Five months after the ocular surgery, the ophthalmic examination revealed an acute anterior unilateral uveitis with small retrodescemetic precipitates on the inferior half of the cornea and choroiditis sequelae. Otherwise, she only reported a chronic cough and the chest CT showed hilar and mediastinal lymphadenopathy. A fine needle biopsy was performed and confirmed the diagnosis of sarcoidosis. She received topical steroids with initial improvement but, at last visit, in August 2016, she had a decreased visual acuity at 20/500 and a papilledema.

***Case 11***

A 58-year-old Caucasian woman presented with a unilateral posterior and intermediate uveitis four months after a YAG capsulotomy. The clinical features included a papilledema and a CSME associated with a conjunctival nodule. The chest X-ray and the chest CT scan showed hilar and mediastinal lymphadenopathy. The diagnosis of sarcoidosis was further confirmed by a conjunctival biopsy and the patient received topical steroids. At last visit, in October 2016, the examination showed a recurrent CSME and the patient was treated with a dexamethasone intravitreal implant.
